# Herbal medicine use among patients with viral and non-viral Hepatitis in Uganda: prevalence, patterns and related factors

**DOI:** 10.1186/s12906-020-02959-8

**Published:** 2020-06-03

**Authors:** Sara Nsibirwa, Godwin Anguzu, Sam Kamukama, Ponsiano Ocama, Joan Nankya-Mutyoba

**Affiliations:** 1 Infectious Disease Institute, College of Health Sciences, Makerere University, Kampala, Uganda; 2 School of Public Health, College of Health Sciences, Makerere University, Kampala, Uganda

**Keywords:** Complementary and alternative medicine, Hepatitis B, Hepatitis C, Herbal medicine

## Abstract

**Background:**

There is some evidence that patients with liver diseases commonly use complementary and alternative therapies to address general and liver-disease specific health concerns. The purpose of this study was to assess and describe prevalence, patterns and related factors of herbal medicine use among adults diagnosed with viral and non-viral hepatitis in Kampala, Uganda.

**Methods:**

A cross-sectional study was conducted on 310 adult patients attending the gastrointestinal clinic in Mulago hospital referral hospital in Kampala. Data on prevalence, types and reasons for herbal medicine use was collected using standardized questionnaires and focus group discussions. Modified Poisson regression analyses were used to examine factors related to use.

**Results:**

Usage of various herbal remedies within 12 months prior to April 2018 was reported by 46.1% (143/310) of patients with 27.3% (39/143) of these reporting having used conventional and herbal therapies concurrently. Herbal remedies were used to treat various health conditions including hepatitis. Patients with hepatitis C virus infection (PRR = 1.16, *p* = 0.02) compared to those with hepatitis B virus infection, and those who believed that it was safe to use herbal and conventional therapies concurrently (PRR = 1.23, *p* = 0.008) had higher prevalence odds of herbal medicine use. Conversely, patients who had been newly diagnosed with hepatitis (PRR = 0.69, *p* = 0.03) compared to those who had been diagnosed more than one-year prior, had lower prevalence odds of herbal medicine use. Various types of local herbs were reported as most commonly used however most patients did not know the ingredients of commercially prepared herbal therapies.

**Conclusion:**

A high prevalence of herbal medicine use was found among newly-diagnosed patients and patients with hepatitis C more likely to use herbal remedies after adjusting for other factors. Usage was influenced by the belief that herbal medicine is safe and effective. Health workers need to consistently elicit information about herbal remedy use. Research is needed on benefits, adverse effects and outcomes in patients who use herbal remedies to treat primary liver diseases in order to facilitate evidence of efficacy and product safety.

## Background

Hepatitis viruses are the most common cause of hepatitis in the world but other infections, substances such as alcohol and drugs, and autoimmune diseases can also cause hepatitis.

Hepatitis B virus (HBV) infection causes significant global burden of disease with 257 million chronically infected [1]. Hepatitis C virus (HCV) infection also poses a significant disease burden globally with an estimated 71 million people reported to have chronic infection [2]. Both infections account for 1.3 million deaths of mortality from cirrhosis and liver cancer per year [3] despite availability of treatment. In Uganda, the most commonly diagnosed forms of hepatitis are caused by HBV with a prevalence ranging from 10 to 25% [4] and alcohol liver disease reported in 10% of the population [5].

As in many other places globally, it has been observed that conventional medical care co-exists with traditional medical care systems in Uganda. Patients may use medicine from one system exclusively or they may acquire medicine from each health system and use it simultaneously or sequentially hence the medical pluralism noted among patients [6]. Due to the high costs of treatment for HCV, the long-term treatment required for HBV infection and treatment-associated adverse effects, limited treatment options for drug-induced liver inflammation(DILI) and alcoholic hepatitis, many patients choose either to supplement antiviral medications with herbal products or to reject conventional therapy altogether and instead rely solely on herbal medicine as an alternative form of therapy [7, 8].

Patients with chronic liver disease have been found to use non-conventional therapies frequently [9, 10] with surveys done in the USA suggesting that the frequency of herbal medicine use among patients with chronic liver disease ranges between 40 and 50% [8] . High rates of herbal use have been found in other populations of patients with liver diseases [11], including only patients with hepatitis C or hepatitis B, or drawing patients from a single institution [12, 13]. Although users of herbal medicines believe they are safer, evidence of toxicity to the liver from using herbal remedies has been documented [14, 15].

There is limited data on herbal medicine use among populations affected by viral and non-viral hepatitis in sub-Saharan Africa. Although several studies have described herbal medicine use in USA [16, 17], in Uganda, few have studied use among a population with pre-existing liver disease. Most studies on herbal use in Uganda and other sub-Saharan countries have focused on populations with HIV and diabetes and there is a paucity of literature on the prevalence and rationale of herbal medicine use among patients with liver disease in sub-Saharan Africa. Specific research to quantify and describe the extent of use among patients with liver diseases is still lacking and little is known about the use of herbal medicine among patients who have been diagnosed with hepatitis in Uganda. A study in rural Rakai examined the relationship between herbal medicine use and liver disease and found an association with liver fibrosis [18]. There is a lack of information on prevalence, patterns and reasons for use, and whether users are aware of possible side effects and complications. This study therefore sought to determine the prevalence of herbal medicine use, explore types of herbal medicines used, reasons for use and determine the factors associated with use among adults with viral and non-viral hepatitis.

## Methods

### Study design and setting

The study was conducted within the gastroenterology clinic of Mulago hospital, a tertiary teaching and National Referral Hospital, in Kampala; which handles up to 100 adult patients weekly on average; with either luminal gastrointestinal issues or liver/pancreatic conditions. The study specifically targeted patients who had a known diagnosis of liver inflammation due to different causes such as hepatitis B virus, hepatitis C virus, drug-induced and alcohol-induced inflammation as shown on their medical records. Data was collected using standardized questionnaires followed by focus group discussions (FGDs) on a sample of patients. The questionnaires used were modified from the International Questionnaire to Measure Use of Complementary and Alternative Medicine (I-CAM-Q) [19];.

### Sample selection

The sample size was calculated based using the Leslie-Kish formula below;

$n=\frac{z^2 pq\ }{e^2}$ at a 95% confidence level, *with an* estimated proportion of herbal medicine use among patients with hepatitis of 23% based on a previous study [20], and 5% precision rate. After adjusting for a non-response rate of 10%, a total sample size of 310 patients was determined and recruited using systematic sampling.

To get the full sample size, approximately 30 patients were interviewed weekly between April and June 2018. To get the sampling interval, 80, which is the average number of hepatitis patients seen weekly was divided by the required number of patients weekly [21] to get an interval of two, therefore every other individual on the log of patients attending the clinic was approached for recruitment. The starting number on the patient log was chosen randomly each week to ensure that all potential patients had an equal chance of selection. Four FGDs, homogenous by gender and purposively sampled, were conducted. Eligibility criteria included consenting adults aged 18 years and above who were diagnosed with either hepatitis B, hepatitis C, alcoholic hepatitis or drug-induced hepatitis. Patients with impaired mental capacity to provide coherent and reliable information were excluded from participating in the study.

### Data collection

Research assistants were trained to administer structured questionnaires. Data collected included socio-demographic factors (age, gender, ethnicity, religion, education level, employment status, type of occupation, residence); use of herbal medicine (ever used, duration of use, use within the last 12 months), the forms, mode of administration and frequency of use of herbal medicine, timing (timing with respect to the diagnosis of hepatitis-categorized as before or after, timing with respect to how often it was used, timing with respect to concurrent use of herbal medicine with conventional medicine) source (the source of the herbal medicine and the source of information about the herbal medicine), clinical factors (duration of illness, diagnosis, type of treatment, duration of treatment) and other factors expected to influence use of herbal medicine (accessibility, perceived benefits from herbal medicine and perceived side effects from herbal medicine). (See survey questionnaire in Additional file 1 attached).

In addition to collecting survey data, the investigators collected data via focus groups. Following each interviews, the research assistants asked for volunteers to participate in focus group discussions to identify how groups of patients think about behaviors and practices linked to herbal medicine use, to explore the reasons for the use of herbal medicine and to examine the social, cultural and economic factors associated with the use of herbal medicine.

Four FGDs were held; two with female patients and two with male patients. Each group was comprising 7–9 patients, homogenous by sex and not widely variable by age. An FGD guide was used to moderate the discussions on the reasons for the use of herbal medicine [22].

### Definitions

The use of herbal medicine was investigated at three levels: ever used, use within 12 months prior to April 2018 and use at the time of the study. Patients who had used herbal medicine at least once in the period of 12 months before April 2018 were classified as herbal medicine users; those who had used herbal medicine at least once in their lifetime but not during the previous 12 months were classified as herbal medicine exposed while those who reported never having used it in their lifetime were classified as non-users.

### Statistical analysis

Data was analyzed using STATA 13.0 (StataCorp. 2013. Statistical Software: Release 13. College Station, TX: StataCorp LP.). Modified Poisson regression analyses [23] were done to determine the factors associated with the use of herbal medicine. Prevalence rate ratios, with 95% confidence interval (CI) were examined to determine the strength of association.

### Analysis of qualitative data

Data from discussions was analyzed immediately following collection. Conclusions were made using thematic analysis.

## Results

The background characteristics of the patients are presented in Table 1 while the characteristics of the patients in the FGDs are presented in supplementary table 1; Additional file 2. The study had 310 patients with a median age of 30 (IQR^1^ 17–73). Majority were aged 25–34 years, male, and residing in urban areas. Most patients were either Catholic (41.6%) or Protestants (38.1%) by religion and had attained secondary level of education as their highest level of education. Over 60% reported being currently employed at the time of the interview, with the larger proportion employed in the informal sector.

**Table 1:** Baseline characteristics of 310 individuals diagnosed with hepatitis attending the gastroenterology clinic in Mulago National Referral Hospital between April and June 2018

Variable	Variable	Frequency(*N* = 310)	Percentage (%)
Age groups	18–24	77	24.8
	25–34	100	32.3
	35–44	74	23.9
	45–54	47	15.2
	≥55	12	3.9
Gender	Male	171	55.2
	Female	139	44.8
Area of residence	Rural	59	19.0
	Urban	251	81.0
Region of origin	Foreign	16	5.2
	Central	129	41.6
	Western	59	19.0
	Northern	46	14.8
	Eastern	60	19.4
Religion	Protestant	118	38.1
	Roman Catholic	129	41.6
	Islam	30	9.7
	Others^a^	33	10.6
Education status	Not educated	4	1.3
	Primary	90	29.0
	Secondary	145	46.8
	Post-secondary^b^	71	22.9
Current employment	No	123	39.7
	Yes	187	60.3
Type of occupation	Formal	60	19.4
	Informal	148	47.7
	Not employed	102	32.9
Current diagnosis	HBV^c^	289	93.2
	HCV^d^	12	3.9
	HBV & HCV	4	1.3
	Drug-induced	3	1.0
	Alcoholic hepatitis	2	0.6
Duration of illness	Less than 6 months	169	54.5
	6–12 months	67	21.6
	13–24 months	34	11.0
	> 24 months	40	12.9
On treatment for hepatitis	No	148	47.7
	Yes	162	52.3
Kind of treatment	Conventional only	150	48.4
	Herbal only	4	1.3
	Conventional&Herbal	8	2.6
	Not on treatment	148	47.7
Duration of treatment	Less than 1 month	37	11.9
	1–6 months	79	25.5
	7–12 months	24	7.7
	13–24 months	16	5.2
	> 24 months	6	1.9
	Not applicable	148	47.7

^a^Others included members of the Pentecostal, Seventh-Day Adventists and Jehovah Witness faiths

^b^Post-secondary included both tertiary and vocational levels

^c^HBV- Hepatitis B virus

^d^HCV-Hepatitis C virus

Over 90% of the patients were diagnosed with hepatitis B and majority of the patients had been diagnosed less than 6 months before participating in the study. Forty-eight percent of the patients were not on any form of treatment for their liver disease while of those who were on treatment, about 72% had been on treatment for less than 6 months.

### Prevalence of herbal medicine use

The use of herbal medicine was investigated at three levels: ever used during lifetime, use within the last 12 months and use at the time of the study.

Lifetime exposure to herbal medicine was reported in 174/310 (56.1%) patients while herbal medicine use within the last 12 months was reported by 143/310 (46.1%) patients.

About 11.9% (37/310) of the patients were reportedly using herbal medicine at the time of the study.

The patients who reported “never used” of herbal medicine were asked to give reasons for never having used it. The main reasons given by patients for non-use of herbal medicine included: lack of trust in the effectiveness of herbal medicine generally (22.8%), lack of knowledge about herbal medicine (16.2%), a lack of interest in using herbal medicine (16.2%), bad taste of herbal medicine (10.3%), easier access to hospitals (8.1%) and lack of trust in the safety of herbal medicines (5.9%).

### Reasons for the use of herbal medicine

The main reasons given for use of herbal medicine in the group discussions were related to convenience, availability, cost, cultural norms and aggressive marketing strategies used by herbal medicine retailers. All these were seen to increase accessibility for the population to herbal medicine therefore leading to increased utilization.

One of the main reasons given as to why herbal medicine was used despite the availability of conventional treatment was the easy accessibility and availability of herbal therapies and herbal practitioners compared to conventional health facilities.

“…Those who sell herbal medicine are everywhere, they even come to one’s door, even in the market. At least one does not get disappointed as it is in the hospital where one is told that the doctor has not yet come, yet one has come from afar and then has to wait the whole day. It is easier to find a herbalist nearby so I can get back home quickly...” (Male, FGD 1).

Several patients also noted that the cheaper cost of herbal therapies as a reason for use of herbal medicine in the group discussions however they concluded that whereas the cost of herbal treatment for simple ailments such as cough and malaria is cheap, herbal concoctions used to treat chronic diseases such as hepatitis end up being expensive due to the long-term use.

Another reason given for use of herbal medicine was that herbal medicine has been used for many years and is a part of the culture hence the reason for its use especially for conditions such as pregnancy. Many of the patients expressed dismay that health workers have a negative attitude towards herbal medicine despite the fact that the knowledge of herbal medicine has been passed down from past generations.

“…Our ancestors used these herbs without getting any problems, all the women in the past used herbs to give birth properly. How come they did not get liver diseases? They knew what was dangerous and they taught it to their children. Even now, we know which herbs are dangerous and which ones are useful. However, you see these doctors tell us that we should not use any herbs. That is not good, because it is a part of our culture and has helped our people for many years. If herbs were bad, then all our people would have died by now...” (Female, FGD 3).

### Indications of herbal medicine use

**Fig. 1:**
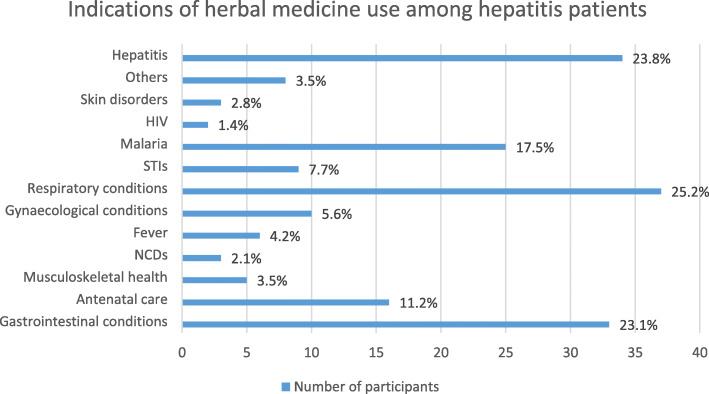
Indications of herbal medicine use among hepatitis patients. The 143 patients who reported use of herbal medicine were asked what conditions they were treating with the herbal medicine. The different bars indicate the frequency of reporting of an indication of use of herbal medicine. However, some patients were using herbal medicine for more than one indication and had multiple responses. Common respiratory conditions were the most frequently reported indications followed by hepatitis and other gastrointestinal conditions. HIV-Human Immunodeficiency Virus; NCDs- Non-Communicable Diseases

Among patients who used herbal medicine within the last 12 months, 34/143(23.8%) were using it to treat hepatitis while 109/143 (76.9%) were using it for various health conditions which are shown in Fig. 1.

This was also confirmed in all the focus groups sessions where patients reported that either they or other patients they knew who had been diagnosed with hepatitis used herbal medicines, not only to treat conditions of the liver but a range of other medical conditions. Twenty-six percent (37/143) reported using herbal medicine for treatment of respiratory conditions such as cough, asthma and the common cold, 33/143 (23.1%) for gastrointestinal conditions including abdominal pain, abdominal discomfort and diarrhea, 25/143(17.5%) for malaria, 16/143(11.2%) for antenatal care and 6/143(4.2%) for fever.

Other indications reported included sexually transmitted infections (7.7%), skin diseases (2.8%) non-communicable diseases(NCDs) such as diabetes and hypertension (2.1%), joint and bone health (3.5%), gynecological conditions such as infertility, post abortion care (5.6%) and erectile dysfunction (1.4%). Conditions such as allergies, epilepsy, general body weakness, kidney stones and alcohol dependence which were reported by only a few patients were reported under other indications on Fig. 1.

### Profile and patterns of herbal medicine use

The most commonly used herbal therapies have been described in Table 2. The majority of the patients reported that while they did know the types of herbal medicines which they picked themselves or get from friends or family members, they did not know which exact herbs are part of the herbal preparations obtained from herbalists and herbal medicine retailers.

**Table 2:** Commonly used herbs by patients diagnosed with viral and non-viral hepatitis in Kampala, Uganda

Local name of herb	Scientific name	Indication	Preparation
Kigagyi	*Aloe vera*	Fever, skin infections, wounds	Boiled and drank as tea/juice, raw sap applied to skin
Ebombo	*Memodica feotida*	Body odour	Ground and applied to body as paste
Kamunye	*Hoslundia opposita*	Wounds, skin infections	Crushed leaves applied directly
Mubiri	–	Anorexia, for weight gain	Juice drank orally
Enjaga	*Cannabis sativa*	Pain relief, antidepressant	Juice drank
Ekiyondo	*Kalanchoe glaucescens*	Nasal congestion, common cold	Crushed and drunk as tea, nasal drops
Mululuza	*Vernonia Amygdalina*	Fever, Malaria	Leaves boiled for drinking as tea
Emumbwa	*Clay soil*	Pregnancy, STIs, Joint aches, abdominal discomfort, Erectile dysfunction, to induce ovulation	Ground in water and drunk Pastes smeared on body
Ekifumufumu	*Leonotis nepetifolia*	Menorrhagia, abdominal pain, muscle aches	Leaves boiled for drinking, smeared
Ssere	*Bidens pilosa*	Goitre, wounds	Leaves crushed and juice applied to wounds
Muyembe	*Mangifera indica*	Cough	Leaves boiled and drunk as tea
Musaayi	*Hibiscus acetosella*	Anemia	Crushed and boiled for drinking
Entangawuzi	*Zingber officinale*	Cough, flu	Chewed whole or boiled in tea

Majority of the patients reported using a liquid form of herbal medicine, with 100/143(69.9%) patients reporting oral administration in form of teas or juices. Herbal medicine retailers and herbalists were reported as the more common sources of herbal medicine with each having 55/143(38.5%) of herbal medicine users reporting them as their sources, 30/143 (21%) obtained the herbal medicine from friends and family members, and 18/143 (12.6%) reporting that they picked the herbal medicine themselves.

The largest proportion of herbal medicine users 109/143 (76.2%); were influenced to do so by family members to use herbal medicine. This was followed by media: television, radio and newspaper advertisements, which influenced 75/143 (52.5%). The patients’ other sources of information about herbal medicine included their friends 34/143 (23.8%); herbal practitioners 13/143 (9.1%); and community elders 8/143 (5.6%).

### Period of time of herbal medicine use

With regards to the period of use, 97/143(67.8%) of herbal medicine users reported that they were using herbal medicine prior to being diagnosed with any form of hepatitis; 91/143(63.6%) reported that they started using herbal medicine after they had been diagnosed with any form of hepatitis; 37.4% of these (34/91) started using the herbal therapies for the treatment of hepatitis with slightly over half (19/34) of these starting treatment within a period of 1 week following diagnosis.

None of the patients reported initiation of herbal therapies for hepatitis treatment more than 6 months after diagnosis.

### Concurrent use of herbal medicine with conventional prescription treatment

Same-day use of both herbal and conventional treatment was reported by 39/143(27.3%). Of these, 13/39 (33.3%) were using both forms of medicine concurrently to treat hepatitis.

The majority of herbal medicine users (129/143; 90.2%) used their herbal therapies intermittently, and 91/143 (63.6%) of users reported using their herbal treatments more than once a week. The profile and patterns of herbal medicine use are further summarized in supplementary table 2. (see supplementary table 2, Additional file 3).

Majority of the group patients concluded that it is not safe to use both kinds of therapies concurrently because they might interact thereby harming the body and leading to side effects such as skin rashes, blindness and organ damage such as kidney and liver damage as exemplified in the statement,

“Yes, it is not safe because one type of drug can reduce the strength of the other and the patient will end up not getting cured at all. It is better to first try one and then try the other.” (Male, 50, FGD2).

### Communication from health workers about herbal medicine use

Only 42%(60/143) of the patients using herbal medicine could recall any health-worker ever asking them about use of herbal medicine, with the majority reporting that they were discouraged from using it and advised to use conventional treatment instead. Only 40.6% (58/143) of users reported ever starting a conversation with their health-worker about herbal therapies.

The main reasons given by patients for not talking to health-workers about herbal medicine included a fear of being discouraged from using herbal medicine (10.2%), 37% of the patients did not find it necessary to talk about herbal medicine and another 10% stated that they had never been asked about it.

This was confirmed in the group discussions which concluded that when health workers do talk about herbal medicine, they mainly discourage use without expounding on any effects that herbal medicines may have.

### Factors associated with herbal medicine use

The following variables were significantly associated with herbal medicine use: education status, duration of illness, current diagnosis, belief that herbal treatment is completely safe and a belief that concurrent use of herbal and conventional treatment is safe as shown in Table 3.

**Table 3:** Factors associated with the use of herbal medicine among patients diagnosed with hepatitis

Factor	Total *N* = 310	Users *n* = 143(%)	Non-users *n* = 167(%)	Unadjusted PRR(95%CI)	*p*-value
Age groups					
18–24	77	21 (14.7)	56 (33.5)	1	1
25–34	100	51 (35.7)	49 (29.3)	1.09 (0.87–1.37)	0.45
35–44	74	31 (21.7)	43 (25.8)	0.97 (0.74–1.27)	0.84
45–54	47	32 (22.4)	15 (9.0)	1.14 (0.91–1.44)	0.26
≥55	12	8 (5.6)	4 (2.4)	1.02 (0.71–1.49)	0.88
Gender					
Male	171	77 (53.9)	94 (56.3)	1	1
Female	139	66 (46.2)	73 (43.7)	0.92 (0.79–1.06)	0.24
Residence					
Rural	59	33 (23.1)	26 (15.6)	1	1
Urban	251	110 (76.9)	141 (84.4)	1.09 (0.91–1.31)	0.33
Education status					
None	4	3 (2.1)	1 (0.6)	1	1
Primary	90	55 (38.5)	35 (21.0)	0.81 (0.72–0.91)	**< 0.001**
Secondary	145	56 (39.2)	89 (53.3)	0.82 (0.74–0.92)	**0.001**
Post-secondary	71	29 (20.3)	42 (25.2)	0.83 (0.71–0.96)	**0.015**
Current Diagnosis					
Hep B	289	130 (90.9)	159 (95.2)	1	1
Hep B & C	4	1 (0.7)	3 (1.8)	0.62 (0.15–2.48)	0.49
Hep C	12	7 (4.9)	5 (3.0)	1.23 (1.14–1.33)	**< 0.001**
Others^a^	5	5 (3.5)	–	1.23 (1.14–1.33)	**< 0.001**
Time since diagnosis					
< 6 months	169	81 (56.6)	88 (52.7)	1	1
6–12 months	67	31 (21.7)	36 (21.6)	0.96 (0.82–1.11)	0.56
13–24 months	34	14 (9.8)	20 (12.0)	0.68 (0.48–0.94)	**0.02**
> 24 months	40	17 (12.0)	23 (13.8)	0.76 (0.57–0.99)	**0.04**
Herbal medicine causing AEs^b^					
No	172	110 (76.9)	62 (37.1)	1	1
Yes	76	23 (16.1)	53 (31.7)	0.79 (0.62–1.01)	**0.06**
Not sure	62	10 (7.0)	52 (31.1)	1.06 (0.87–1.30)	0.53
Safe to use both					
No	192	53 (37.1)	139 (83.2)	1	1
Yes	111	85 (59.4)	26 (15.6)	1.19 (1.02–1.39)	**0.03**
Not sure	7	5 (3.5)	2 (1.2)	1.36 (1.18–1.56)	**< 0.001**
Higher cost of treatment					
Conventional	194	98 (68.5)	96 (57.5)	1	1
Herbal	104	44 (30.8)	60 (35.9)	0.94 (0.81–1.09)	0.42
Not sure	12	1 (0.7)	11 (6.6)	0.29 (0.05–1.61)	0.16
Communication from health workers					
No	212	83 (58.0)	129 (77.3)	1	1
Yes	98	60 (42.0)	38 (22.8)	1.02 (0.89–1.18)	0.74
Total	**310**	**143 (100%)**	**167 (100%)**		

^a^Others includes both alcohol-induced hepatitis and drug-induced hepatitis

^b^AEs- adverse effects

The patients who had attained any form of education were less likely to use herbal medicine compared to those who were not educated however on adjusting for other factors, the education status was not found to be a significant factor in determining herbal medicine use.

Patients who had been diagnosed with hepatitis for a duration greater than 1 year; between 13 and 24 months (PRR^2^ 0.68,95% CI 0.48–0.94, p 0.02); longer than 24 months (PRR 0.76, 95% CI 0.57–0.99, p 0.04) before the study were also found to be less likely to use herbal therapies compared to those who had been diagnosed with hepatitis for a duration of less than 1 year before adjusting for other factors.

There was also a significant difference in the use of herbal medicine according to the type of hepatitis with which patients had been diagnosed. Patients who were diagnosed with hepatitis C (PRR 1.23, 95% CI 1.14–1.33, *p* < 0.001) and those diagnosed with other forms of hepatitis including alcoholic hepatitis and drug-induced hepatitis (PRR 1.23,95% CI 1.14–1.33, p < 0.001) were more likely to use herbal therapies compared to those who were diagnosed with hepatitis B before adjusting for other factors.

The perceived safety of herbal therapies was also shown to have a significant difference in use with the patients who believed that herbal medicines cause adverse effects (AEs) less likely to use herbal therapies compared to those who believed that herbal medicines are harmless (PRR 0.79,95% CI 0.62–1.01, p0.06) before adjusting for other factors. Similarly, patients who believed that it is safe to use both herbal and conventional therapies concurrently (PRR 1.19, 95% CI 1.02–1.39, p0.03) or who were not certain (PRR 1.36, 95% CI 1.18–1.56, *p* < 0.001) were more likely to use herbal medicines compared to those who believed that it was unsafe to use both forms of therapies concurrently before adjustment.

No significant differences in prevalence of herbal medicine use was found according to age, sex, employment status, employment type, religion, area of residence, religion, accessibility, use of conventional treatment, cost of treatments and communication from health-workers. There was no significant difference in the prevalence odds of herbal medicine use between patients who reported that herbal medicine was more expensive compared to those who reported that conventional medicine was more expensive. Similarly, there was no significant difference in prevalence odds of herbal medicine use between patients who reported easier accessibility to either healthcare practitioners or herbal medicine practitioners.

After adjusting for demographic and clinical factors, patients who were more likely to use herbal medicine were those who were diagnosed with chronic hepatitis C infection (PRR 1.16; 95% CI 1.07–1.31, *p* = 0.02), compared to those with hepatitis B and those who believed that it was safe to concurrently use both herbal and conventional medicine (PRR 1.32; 95% CI 1.10–1.59, *p* = 0.003) compared to those who thought it was not safe to use both therapies.

Patients who had been diagnosed with any form of hepatitis between 1 to 2 years prior to the study (PRR 0.69; 95%CI 0.49–0.98, *p* = 0.03) and those who had been diagnosed over 2 years’ prior (PRR 0.77; 95% CI 0.59–0.98, *p* = 0.05) were less likely to use herbal medicine. This is shown in Table 4.

**Table 4:** Results of multivariate analysis of factors associated with herbal medicine use among patients diagnosed with hepatitis

Factor	Categories	Adjusted PRR	*p*-value
Socio-demographic			
Age groups	18–24	1	1
	25–34	1.10 (0.88–1.34)	0.41
	35–44	0.99 (0.75–1.29)	0.95
	45–54	1.18 (0.93–1.49)	0.17
	≥55	1.17 (0.79–1.76)	0.43
Gender	Male	1	1
	Female	0.94 (0.82–1.06)	0.31
Education status	None	1	1
	Primary	0.96 (0.83–1.10)	0.54
	Secondary	1.00 (0.88–1.15)	0.95
	Post-secondary	1.05 (0.89–1.25)	0.52
Clinical factors			
Current Diagnosis	Hep B	1	1
	Hep B & C	0.52 (0.13–2.08)	0.36
	Hep C	1.16 (1.07–1.31)	**0.02**
	Others	1.04 (0.92–1.17)	0.52
Time since diagnosis	< 6 months	1	1
	6–12 months	0.96 (0.84–1.10)	0.52
	13–24 months	0.69 (0.49–0.98)	**0.03**
	> 24 months	0.77 (0.59–0.98)	**0.05**
On conventional treatment	No	1	1
	Yes	0.99 (0.87–1.15)	0.98
Safety profile			
Safe to use both	No	1	1
	Yes	1.23 (1.05–1.43)	**0.008**
	Not sure	1.32 (1.10–1.59)	**0.003**

## Discussion

A high prevalence of herbal medicine use among viral and non-viral hepatitis patients in this sub-Saharan African setting was reported; similar to that observed in a similar adult population diagnosed with viral hepatitis in the US [8]. Usage was independent of socio-demographic factors and was not related to concurrent use of prescription medicine for hepatitis. Conventional and traditional medicine systems run parallel in Uganda, with patients switching from one form to another depending on which they believe is most suitable for a particular condition. Conventional medicine is expensive and relatively not accessible, compared to traditional forms of treatment. It is, therefore, not surprising that most patients resort to herbal therapies. Similar to findings of previous studies globally [24–30], our study found evidence to suggest that cultural attitudes and personal beliefs towards health and life strongly play a role in the motivation for and are more compatible with herbal medicine use which goes beyond sociodemographic determinants such as educational status, occupation and age.

Patients who were newly diagnosed up to 6 months were more likely to use herbal medicine, possibly because that they had not yet been well sensitized about the management of hepatitis and the potential dangers of using herbal medications in liver disease or that they had been using them prior to diagnosis. Namuddu et al. in 2011 also showed that newly diagnosed HIV patients on anti-retroviral therapy were more likely to use herbal therapies [21].

Cost was not been found to be a predictor of use in this study; consistent with other studies showing that cost only indirectly influences the use of alternative medicines; with accessibility and availability playing a bigger role [26, 28]. Hepatitis B infected patients on antiviral treatment in Uganda are treated at no cost; however, there are limited treatment options for hepatitis C with the available antiviral medication having prohibitive costs; likely playing a role in encouraging use of alternative therapies in this subset; similarly shown in a study by Ferruci et al. in 2010 [20].

Effective therapies for alcoholic-induced hepatitis and DILI are still limited, therefore influencing the use of herbal therapies which is also consistent with other findings [31].

The majority of herbal medicine users believed that herbal medicines were safe and concurrent use of both conventional and herbal medicine was perceived safe as well; congruent with other studies [28, 32]. Traditional use over many generations, without known negative effects, in addition to being natural, implies that herbal medicines are completely safe. However, herbal medicine has been known to cause herb-induced liver injury(HILI) [18, 33, 34] when used alone or in combination with prescription medicines [35] and it is therefore imperative for health workers to educate patients on the potential dangers of herbal medicine use. This study did not explore whether liver injury might have occurred as a result of use of herbal medicine because despite different studies demonstrating evidence of herbal hepatotoxicity, there are challenges in establishing a diagnosis of HILI when the biochemical abnormalities of chronic liver disease already exist.

Most of our patients are not willing to disclose to health workers that they are using herbal medicine fearing discouragement and victimization, but also health-workers do not routinely talk to patients about this, which was consistent with studies in Australia and South Africa [36]. Given that adults seek the services of conventional health workers either after or concurrently with those of alternative medicine providers, health-workers have a unique opportunity to advise patients on the use of complementary and alternative medicine (CAM) and should do so without judgement and reproach.

A majority of patients rely on family members and friends for information about herbal medicine as well as mass media. Personal referral and recommendation for CAM has been previously identified as a powerful motivator for use [37, 38]. Extensive advertising of services and products by herbal practitioners can expose the population to unsubstantiated claims of disease management making this a pitfall as a source of knowledge for patients [39, 40]. Herbal medicine messages in the mass media should be regulated to ensure population protection.

This study has a number of limitations which should be considered. Interpretation of our findings was based on self-report of herbal medicine use by the patients and prone to the effects of recall bias; which was mitigated by use of qualitative interviews to triangulate findings. Different understandings of the term ‘herbal medicine’ used in the interviews might have influenced the responses given but this influence was minimized by the interviewers elaborating on the questions and probing particularly for the use of local medicine and herbal extracts.

## Conclusion

Our study provides insights into the use of herbal medicine by patients with viral and non-viral hepatitis. We showed that herbal medicine is used by a large proportion of patients, it is easily accessible and health care workers rarely discuss herbal medicine use during the process of providing care. Perceptions of safety of herbal medicines, hepatitis C diagnosis and recent diagnosis positively influence herbal medicine use.

To warrant patients’ safety, dissemination of knowledge about herbal medicines both to health workers and as information for the general public can facilitate the conversation on potential risks and benefits thus promoting rational use. Health workers should regularly engage in conversations with their patients about their herbal medicine usage. Regulation of herbal medicine practitioners and products should be carried out and a streamlined policy on alternative healthcare in line with the population’s cultural practices in Uganda should be developed. Findings suggest a need for clinical trials to determine the safety and efficacy of CAM products for the treatment of hepatitis B and C in comparison to conventional antiviral treatment.

## Supplementary information

**Additional file 1:** This contains the survey questions administered to the study participants.

**Additional file 2: Supplementary Table S1.** Baseline characteristics of the Focus Group Discussions. This contains information on the baseline characteristics of the focus group discussions including age and gender.

**Additional file 3: Supplementary Table S2.** Profile of herbal medicine use among patients with hepatitis. This contains information on the profile of use of herbal medicine as collected from the participant interviews.

## Data Availability

The datasets used and/or analysed during the current study are available from the corresponding author on reasonable request.

## Abbreviations

CAM: Complementary and Alternative Medicine
DILI: Drug-induced liver inflammation
FGD: Focus group discussion
HBV: Hepatitis B virus
HCV: Hepatitis C virus
HILI: Herb-induced liver injury
HIV: Human Immunodeficiency Virus
NCDs: Non-Communicable Diseases
PRR: Prevalence rate ratio
STIs: Sexually Transmitted Infections

## Footnotes

1 IQR- Interquartile range

2 PRR- Prevalence rate ratio

## References

[1] Hepatitis B factsheet. In: World Health Organization:media centre[website]. July 2019 update. http:www.who.int/mediacentre/factsheets/fs204/en/. Accessed 7 Jan 2020.

[2] Global hepatitis report. Geneva: World Health Organization; 2017. Available at http://www.who.int/hepatitis/publications/global-hepatitis-report2017_eng. Accessed July 2018.

[3] Ringehan M, McKeating JA, Protzer U (2017). Viral hepatitis and liver cancer. Phil Trans R Soc B.

[4] Bwogi J, Braka F, Makumbi I, et al. Hepatitis B infection is highly endemic in Uganda: findings from a national serosurvey. African Health Sciences. 2009;9(2):98-108. PMC2707048 19652743

[5] Opio CK, Seremba E, Ocama P, Lalitha R, Kagimu M, Lee WM (2013). Diagnosis of alcohol misuse and alcoholic liver disease among patients in the medical emergency admission service of a large urban hospital in sub-Saharan Africa; a cross sectional study. Pan Afr Med J.

[6] Langlois-Klassen D, Kipp W, Jhangri GS, Rubaale T (2007). Use of traditional herbal medicine by AIDS patients in Kabarole District, western Uganda. Am J Trop Med Hyg.

[7] Strader DB, Bacon BR, Lindsay KL, La Brecque DR, Morgan T, Wright EC (2002). Use of complementary and alternative medicine in patients with liver disease. Am J Gastroenterol.

[8] Stickel F, Schuppan D (2007). Herbal medicine in the treatment of liver diseases. Dig Liver Dis.

[9] Levy C, Seeff LD, Lindor KD (2004). Use of herbal supplements for chronic liver disease. Clin Gastroenterol Hepatol.

[10] Siddiqui U, Weinshel EH, Bini EJ (2004). Prevalence and predictors of herbal medication use in veterans with chronic hepatitis C. J Clin Gastroenterol.

[11] White CP, Hirsch G, Patel S, Adams F, Peltekian KM (2007). Complementary and alternative medicine use by patients chronically infected with hepatitis C virus. Can J Gastroenterol.

[12] Bean P (2002). The use of alternative medicine in the treatment of hepatitis C. Am Clin Lab.

[13] Chang J-M, Huang K-L (2007). Complementary and alternative therapies in the treatment of chronic hepatitis B. Hepatitis B Ann.

[14] Melchart D, Hager S, Albrecht S, Dai J, Weidenhammer W, Teschke R (2017). Herbal traditional Chinese medicine and suspected liver injury: a prospective study. World J Hepatol.

[15] de Boer YS, Sherker AH (2017). Herbal and Dietary Supplement–Induced Liver Injury. Clin Liver Dis.

[16] Kessler RC, Davis RB, Foster DF, Van Rompay MI, Walters EE, Wilkey SA (2001). Long-term trends in the use of complementary and alternative medical therapies in the United States. Ann Intern Med.

[17] Gupta NK, Lewis JH (2008). Review article: the use of potentially hepatotoxic drugs in patients with liver disease. Aliment Pharmacol Ther.

[18] Auerbach BJ, Reynolds SJ, Lamorde M, Merry C, Kukunda-Byobona C, Ocama P (2012). Traditional herbal medicine use associated with liver fibrosis in rural Rakai, Uganda. PLoS One.

[19] Quandt SA, Verhoef MJ, Arcury TA, Lewith GT, Steinsbekk A, Kristoffersen AE (2009). Development of an international questionnaire to measure use of complementary and alternative medicine (I-CAM-Q). J Altern Complement Med.

[20] Ferrucci LM, Bell BP, Dhotre KB, Manos MM, Terrault NA, Zaman A (2010). Complementary and alternative medicine use in chronic liver disease patients. J Clin Gastroenterol.

[21] Namuddu B, Kalyango JN, Karamagi C, Mudiope P, Sumba S, Kalende H (2011). Prevalence and factors associated with traditional herbal medicine use among patients on highly active antiretroviral therapy in Uganda. BMC Public Health.

[22] World Health Organization. How to investigate the use of medicines by consumers / Anita Hardon, Catherine Hodgkin, and Daphne Fresle. World Health Organization. 2004. https://apps.who.int/iris/handle/10665/68840.

[23] Zou G (2004). A modified Poisson regression approach to prospective studies with binary data. Am J Epidemiol.

[24] Lövgren M, Wilde-Larsson B, Hök J, Leveälahti H, Tishelman C (2011). Push or pull? Relationships between lung cancer patients’ perceptions of quality of care and use of complementary and alternative medicine. Eur J Oncol Nurs.

[25] Sirois FM, Gick ML (2002). An investigation of the health beliefs and motivations of complementary medicine clients. Soc Sci Med.

[26] Gyasi RM, Mensah CM, Osei-Wusu Adjei P, Agyemang S (2011). Public perceptions of the role of traditional medicine in the health care delivery system in Ghana.

[27] McLaughlin D, Lui C-W, Adams J (2012). Complementary and alternative medicine use among older Australian women-a qualitative analysis. BMC Complement Altern Med.

[28] Gyasi RM, Asante F, Yeboah JY, Abass K, Mensah CM, Siaw LP (2016). Pulled in or pushed out? Understanding the complexities of motivation for alternative therapies use in Ghana. Int J Qual Stud Health Well Being.

[29] Chandrakumar A, Xavier A, Xavier A, Manakkadiyil A, Reghu A, Thomas L (2016). Implications of traditional medicine in the treatment of Hepatitis a in Kerala. J Tradit Complement Med.

[30] Payyappallimana U. Role of traditional medicine in primary health care: An overview of the perspectives and challenges. Yokohama J Soc Sci. 2009;14:69–72.

[31] Milosevic N, Milanovic M, Turkulov V, Stojanoska M, Abenavoli L, Milic N (2016). May patients with alcohol liver disease benefit from herbal medicines?. Rev Recent Clin Trials.

[32] Holst L, Wright D, Haavik S, Nordeng H (2009). The use and the user of herbal remedies during pregnancy. J Altern Complement Med.

[33] Tilburt JC, Kaptchuk TJ (2008). Herbal medicine research and global health: an ethical analysis. Bull World Health Organ.

[34] Lui CW, Dower J, Donald M, Coll JR (2012). Patterns and determinants of complementary and alternative medicine practitioner use among adults with diabetes in Queensland, Australia. Evid Based Complement Altern Med.

[35] Agbabiaka TB, Wider B, Watson LK, Goodman C (2017). Concurrent use of prescription drugs and herbal medicinal products in older adults: a systematic review. Drugs Aging.

[36] Xue CC, Zhang AL, Lin V, Da Costa C, Story DF (2007). Complementary and alternative medicine use in Australia: a national population-based survey. J Altern Complement Med.

[37] Bennett J, Brown CM (2000). Use of herbal remedies by patients in a health maintenance organization. J Am Pharm Assoc.

[38] Sirois FM, Purc-Stephenson RJ (2008). Consumer decision factors for initial and long-term use of complementary and alternative medicine. Complement Health Pract Rev.

[39] Peacock M, Badea M, Bruno F, Timotijevic L, Laccisaglia M, Hodgkins C (2019). Herbal supplements in the print media: communicating benefits and risks. BMC Complement Altern Med.

[40] Munyaradzi M. Ethical quandaries in spiritual healing and herbal medicine: a critical analysis of the morality of traditional medicine advertising in southern African urban societies. Pan Afr Med J. 2011;10:6. 10.4314/pamj.v10i0.72212. 10.4314/pamj.v10i0.72212 PMC3282931 22187588

