# Supplementary material for: Herbal medicine use among patients with viral and non-viral Hepatitis in Uganda: prevalence, patterns and related factors
Source: BMC Complement Med Ther. 2020 Jun 3;20:169. doi: 10.1186/s12906-020-02959-8 (PMC7268757; doi:10.1186/s12906-020-02959-8)
Supplement: Supplementary file 1 — Additional file 1: This contains the survey questions administered to the study participants. [file 12906_2020_2959_MOESM1_ESM.pdf]

APPENDIX 1: STUDY QUESTIONNAIRE

TITLE: Prevalence and factors associated with use of Herbal Medicines by Adults infected with hepatitis in Mulago Hospital

Introduction I am from the School of Public Health Makerere University conducting this study and I am requesting for a few minutes of your time to ask you some questions. Your responses will be treated as confidential and anonymous. Your participation in this study is voluntary. You have the right to refuse or to withdraw from the study any time. The information from this study will enable health workers to improve the care given to people diagnosed with hepatitis. Thank you.

Date: ………………………. Participant number: ……………….

SECTION I: Socio-demographic characteristics

Sex:

1. Age:
2. Residence:
3. Occupation:
4. Ethnicity:
5. Religion:
6. Highest level of education attained: Primary Secondary University None
7. Are you currently employed?
8. How long ago were you diagnosed with liver disease?
9. Are you on any treatment for the liver?
   If yes, which treatment are you on?
   For how long have you been on treatment?

SECTION II: Questions about herbal medicine use

10. Have you ever used herbal medicine? Yes No

    a) If yes, what form of herbal medicine do you use?

i)Liquid form ii) Solid form e.g. clay stone iii) Smoke form iv) Leaves, roots or barks of trees v) Mixed formulation

b) If no, why not?

(For those who answer No, skip to question 21.)

11. Have you used herbal medicine in the last 12 months?
12. Are you currently using any herbal medicine?
13. How long have you been using it?
14. Where do you get this herbal medicine?
    a) Picked it yourself From a friend/ family member b) From a herbalist c)From a herbal medicine retailer d) Other
15. From whom or where did you learn about herbal medicine and its use?
    a) Family member b) Community elder c) Herbalist d) Friend e) Media (television, radio, newspapers) f) Other:
16. Are you using it to treat Hepatitis?
    b) If not, what are you using it for?
17. Were you using this herbal medicine before your diagnosis of liver disease?
    b) Did you continue using it after you were diagnosed with liver disease?
18. How often do you use it?
    a) Daily b) once a week c) more than once a week d) monthly e) Other
19. How do you use it?
    a) Oral ingestion b) Body smearing/ bathing c) Inhalation of smoke d) Other
20. Do you use it consistently as directed?
    b) Is your use continuous or intermittent?
21. Do you take conventional medicine prescribed in the hospital for Hepatitis treatment?
22. Have you ever used herbal medicine on the same day as you were taking the treatment for hepatitis from the hospital?

    b) If yes, why do you use herbal medicine as well as conventional medicine?

    - The herbs are more effective
    - Side effects of conventional drugs
    - It is cheap
    - Herbs are easily accessible
    - Improves well-being
    - Other (specify)

SECTION III: Questions concerning factors associated with use of herbal therapies

23. Have you experienced any of the following effects while using herbal medicine?

a) Vomiting b) Diarrhea c) Abdominal pain d) Skin rash e) Yellowing of the eyes or skin f) Fever g) Other h) I have not experienced any side effects

24. Has this herbal medicine led to any improvement in your health?

If yes, what improvement have you noticed?

a) Improved health status b) Decreased symptoms of disease c) Other d) I cannot tell if there has been any difference

25. Have you ever talked to your health care worker about the use of herbal medicine?

Yes No I do not remember

If no, why not? If yes, what was his/her response?

26. Has your doctor/nurse ever asked you about herbal medicine use?

Yes No I do not remember

27. In your opinion, do you think it is safe to use both conventional and herbal medicine at the same time? b) If no, please give reasons
28. Do you know whether the use of herbal medicine alone can cause any adverse effects in the body? b) If yes, which ones do you know it to cause?
29. Do you pay for the conventional prescribed treatment for liver disease? (*skip if answer to question 21 is no*)
30. Do you pay for the herbal medicine you use?
31. If yes, is the amount more than what you pay/spend to get conventional treatment?
32. How do you compare the accessibility of herbal medicine providers to conventional health care providers in your community? a) Herbalists are more accessible b) Health care providers are more accessible c) They are equally accessible
33. Do you experience any challenges with accessing conventional treatment for liver disease? If yes, what challenges do you meet? a) Distance b) Cost of treatment high c) No drugs in the hospital/health facility d) Drug side effects e) Unfriendly/rude health workers f) Long waiting time/delays g) Others (specify)

This concludes the interview. Thank you for taking the time to answer our questions.
