# Supplementary material for: Herbal medicine use among patients with viral and non-viral Hepatitis in Uganda: prevalence, patterns and related factors
Source: BMC Complement Med Ther. 2020 Jun 3;20:169. doi: 10.1186/s12906-020-02959-8 (PMC7268757; doi:10.1186/s12906-020-02959-8)
Supplement: Supplementary file 2 — Additional file 2: Supplementary Table S1. Baseline characteristics of the Focus Group Discussions. This contains information on the baseline characteristics of the focus group discussions including age and gender. [file 12906_2020_2959_MOESM2_ESM.docx]

#### **Supplementary Table I: Baseline characteristics of the Focus Group Discussions**

| Characteristic | FGD1 | FGD2 | FGD3 | FGD4 |
| --- | --- | --- | --- | --- |
| Sex | Male | Male | Female | Female |
| Age ranges | 30-56 years | 33-68 years | 25-48 years | 21-54 years |
|  |  |  |  |  |
