# Supplementary material for: Herbal medicine use among patients with viral and non-viral Hepatitis in Uganda: prevalence, patterns and related factors
Source: BMC Complement Med Ther. 2020 Jun 3;20:169. doi: 10.1186/s12906-020-02959-8 (PMC7268757; doi:10.1186/s12906-020-02959-8)
Supplement: Supplementary file 3 — Additional file 3: Supplementary Table S2. Profile of herbal medicine use among patients with hepatitis. This contains information on the profile of use of herbal medicine as collected from the participant interviews. [file 12906_2020_2959_MOESM3_ESM.docx]

Supplementary Table 2: Profile of herbal medicine use among patients with hepatitis

| Item | Frequency(N=143) | Percentage (%) |
| --- | --- | --- |
| Form of herbs used |  |  |
| Liquid | 100 | 69.9% |
| Solid | 1 | 0.70% |
| Pastes | 1 | 0.70% |
| Mixed formulations | 41 | 28.7% |
| How is it administered^[[1]](#footnote-1)^ |  |  |
| Orally | 137 | 95.8% |
| Body smearing/bathing | 16 | 11.2% |
| Smoke inhalation | 1 | 0.7% |
| Source of herbs |  |  |
| Picked by participant | 18 | 12.6% |
| Friend/family member | 30 | 20.9% |
| Herbalist | 55 | 38.4% |
| Herbal medicine retailer | 55 | 38.4% |
| Source of knowledge |  |  |
| Family member | 89 | 62.2% |
| Community elder | 8 | 5.6% |
| Herbalist | 13 | 9.1% |
| Friend | 34 | 23.8% |
| Media | 75 | 52.5% |
| Others | 1 | 0.7% |
| Timing of use |  |  |
| Before hepatitis diagnosis | 97 | 67.8% |
| Started/Continued after diagnosis | 90 | 62.9% |
| How often is it used |  |  |
| Everyday | 45 | 31.5% |
| Once a week | 2 | 1.4% |
| More than once a week | 91 | 63.6% |
| Whenever I get symptoms | 5 | 3.5% |
| Pattern of use^[[2]](#footnote-2)^ |  |  |
| Continuous | 13 | 9.8% |
| Intermittent | 129 | 90.2% |
| Concomitant use with conventional treatment^[[3]](#footnote-3)^ |  |  |
| Yes | 39 | 27.3% |
| No | 39 | 27.3% |
| Do you pay for herbal medicine used |  |  |
| Yes | 119 | 83.2% |
| No | 24 | 16.7% |

1. Some participants had multiple responses [↑](#footnote-ref-1)
2. 1 participant missing information [↑](#footnote-ref-2)
3. 65(45.4%) participants using herbal therapy were not on conventional treatment for hepatitis. [↑](#footnote-ref-3)
